# PDE inhibition in distinct cell types to reclaim the balance of synaptic plasticity

**DOI:** 10.7150/thno.50701

**Published:** 2021-01-01

**Authors:** Ben Rombaut, Sofie Kessels, Melissa Schepers, Assia Tiane, Dean Paes, Yevgeniya Solomina, Elisabeth Piccart, Daniel van den Hove, Bert Brône, Jos Prickaerts, Tim Vanmierlo

**Affiliations:** 1 Department of Neurosciences, European Graduate School of Neuroscience, Biomedical Research Institute, UHasselt, Hasselt University, Hasselt, Belgium.; 2 Department Psychiatry and Neuropsychology, European Graduate School of Neuroscience, School for Mental Health and Neuroscience, Maastricht University, Maastricht, Netherlands.; 3 Tomsk State University, Tomsk, Russia.; 4 Department of Psychiatry, Psychosomatics, and Psychotherapy, University of Wuerzburg, Wuerzburg 97080, Germany.

**Keywords:** neurodegeneration, synapses, phosphodiesterase, cell-signaling, glia-neuron

## Abstract

Synapses are the functional units of the brain. They form specific contact points that drive neuronal communication and are highly plastic in their strength, density, and shape. A carefully orchestrated balance between synaptogenesis and synaptic pruning, *i.e.*, the elimination of weak or redundant synapses, ensures adequate synaptic density. An imbalance between these two processes lies at the basis of multiple neuropathologies. Recent evidence has highlighted the importance of glia-neuron interactions in the synaptic unit, emphasized by glial phagocytosis of synapses and local excretion of inflammatory mediators. These findings warrant a closer look into the molecular basis of cell-signaling pathways in the different brain cells that are related to synaptic plasticity. In neurons, intracellular second messengers, such as cyclic guanosine or adenosine monophosphate (cGMP and cAMP, respectively), are known mediators of synaptic homeostasis and plasticity. Increased levels of these second messengers in glial cells slow down inflammation and neurodegenerative processes. These multi-faceted effects provide the opportunity to counteract excessive synapse loss by targeting cGMP and cAMP pathways in multiple cell types. Phosphodiesterases (PDEs) are specialized degraders of these second messengers, rendering them attractive targets to combat the detrimental effects of neurological disorders. Cellular and subcellular compartmentalization of the specific isoforms of PDEs leads to divergent downstream effects for these enzymes in the various central nervous system resident cell types. This review provides a detailed overview on the role of PDEs and their inhibition in the context of glia-neuron interactions in different neuropathologies characterized by synapse loss. In doing so, it provides a framework to support future research towards finding combinational therapy for specific neuropathologies.

## Introduction

The central nervous system (CNS) consists of different cell types with dedicated functions, *i.e.* neurons and glial cells, which together create and maintain the brain's circuitry. Synapses are contact points between neurons that allow them to communicate with each other and, hence, are vital to proper brain function. The amount of synapses is strictly and dynamically regulated throughout the human lifespan by constant turnover of synapses. Regular formation of synapses is offset by the elimination of synapses that are too weak or in excess 1. In the aging or degenerating brain, this balance shifts towards loss of synapses, leading to a decline in neurological function. Synapse elimination can be influenced by physiological pathways within the neuron itself or by active removal of synapses by surrounding glial cells 2.

Synapse formation and elimination rely heavily on second messengers as effectors 3-5. Among these second messengers, 3'5'-cyclic adenosine monophosphate (cAMP) and 3'5'-cyclic guanosine monophosphate (cGMP) show widespread expression, making them interesting targets in CNS resident cells. Compartmentalization of сAMP and cGMP is mainly determined by localization of macromolecular complexes formed by A-kinase anchor proteins (AKAPs) and G-kinase anchor proteins (GKAPs), respectively 6-8. These anchoring proteins play an essential role by directing localization of cAMP and cGMP-dependent protein kinases (*i.e.* protein kinase A [PKA] and PKG, respectively) close to their substrates (cAMP and cGMP), allowing an adequate physiological response of the cell to a signal. Despite the signaling contribution of cGMP to neuroplasticity, GKAP/PKG signalosomes are not well characterized yet. This review will focus on AKAP/cAMP complexes. Extensive research has demonstrated that elevating cAMP and cGMP levels can drive the CNS towards a neuroprotective environment to prevent damage 9-17. Achieving this elevation is possible through the anabolic pathway via guanylyl cyclase (GC) or adenylyl cyclase (AC), or by blocking phosphodiesterases (PDEs), which hydrolyze and break down cGMP and cAMP.

The physiology of PDEs carries a well-established diversity: 21 genes have been identified, which code for proteins that differ vastly in their regulatory properties and spatiotemporal organization. The 21 genes of the PDE superfamily can be distinguished by their catalytic unit and are grouped in 11 different families (PDE1-PDE11) based on protein structure and regulatory domains. These protein structures affect substrate selectivity (cGMP or cAMP, as shown in **Table 1**) and interactions with mediators of phosphorylation. The different genes (*e.g.*, PDE4A-D) are unique in terms of their genomic organization, including the use of multiple promoters. Divergent isoforms (*e.g.*, PDE4D1-9) exist owing to the existence of multiple promoters and the influence of alternative splicing. Isoforms of PDE4, for instance, can be classified into long, short, or supershort, based on the presence of upstream conserved regions (UCR's). Long isoforms have two of these UCR's, short isoforms have one, and the supershort has a truncated version of one. The presence of different PDE transcripts and their association with anchoring proteins in distinct pathways of cell signaling throughout the CNS leads to the widespread effects of PDE-mediated signaling pathways. These biochemical differences have also comprehensively been elucidated in research studies using *C. elegans* 18, 19. Additionally, these studies have facilitated progress towards specialized drug targeting to influence specific pathways in neurobiology 20-24.

Synapses can differ according to the neurons with which they are made up. Among chemical synapses, two types can be distinguished, *i.e.* excitatory and inhibitory, of which the most prevalent in the mammalian brain are glutamatergic and GABAergic (relying on gamma-aminobutyric acid) synapses. Glutamatergic synapses rely on both metabotropic (mGluRs) and ionotropic glutamate receptors, such as kainite receptors, N-methyl-D-aspartate (NMDA) and α-amino-3-hydroxy-5-methyl-4-isoxazolepropionic acid (AMPA) receptors, of which the latter is responsible for baseline neurotransmission. Importantly, the synapse type dictates its structure. Inhibitory synapses (*e.g.* GABAergic) are mostly formed directly on dendritic shafts. Excitatory synapses (*e.g.* glutamatergic) are usually formed by using dendritic spines, which are small membrane protrusions from dendritic shafts, creating a postsynaptic partner for excitatory neurons 2, 25. The importance of the two types of synapses is readily apparent in the complex interplay of neuronal excitation and inhibition, which dynamically regulates the neuronal circuitry. The tight coordination of these different inputs ensures a constant excitation/inhibition (E/I) ratio. This ratio has previously been described to be of crucial importance for efficient coding of information, while on the single-neuron level, it may safeguard efficient signal transduction and survival 26. A plethora of neuropathologies, including autism, epilepsy, schizophrenia, and Alzheimer's disease (AD), have been linked to a disrupted E/I ratio 25, 27.

Apart from plasticity that is inherent to neuronal functioning, the effect of interaction of synapses with other, external, factors should not be overlooked. Glial cells, responsible for homeostatic balance and support of the neuronal circuit, play a major role in plasticity. Apart from indirectly changing the micro-environment, glial cells have emerged as direct regulators of synapse maintenance and elimination. Endfeet of astrocytes converge on neuronal contact sites to create the tripartite synapse 2. On top of that, microglia interact dynamically with this tripartite synapse using their highly motile processes, leading to the emergence of the term 'quad-partite synapse' 28. Both glial cell types contribute to synapse homeostasis by interacting with synapses directly, as well as indirectly by the release of soluble factors. The interplay between neurons, astrocytes, oligodendrocytes, and microglia involves a broad range of soluble factors, all contributing to the CNS environment and the dynamics of synapse formation or elimination. Moreover, mounting evidence describing crosstalk between microglia and astrocytes further highlights the importance of glial cells in synapse maintenance and plasticity 2, 29, 30.

**Figure 1:**
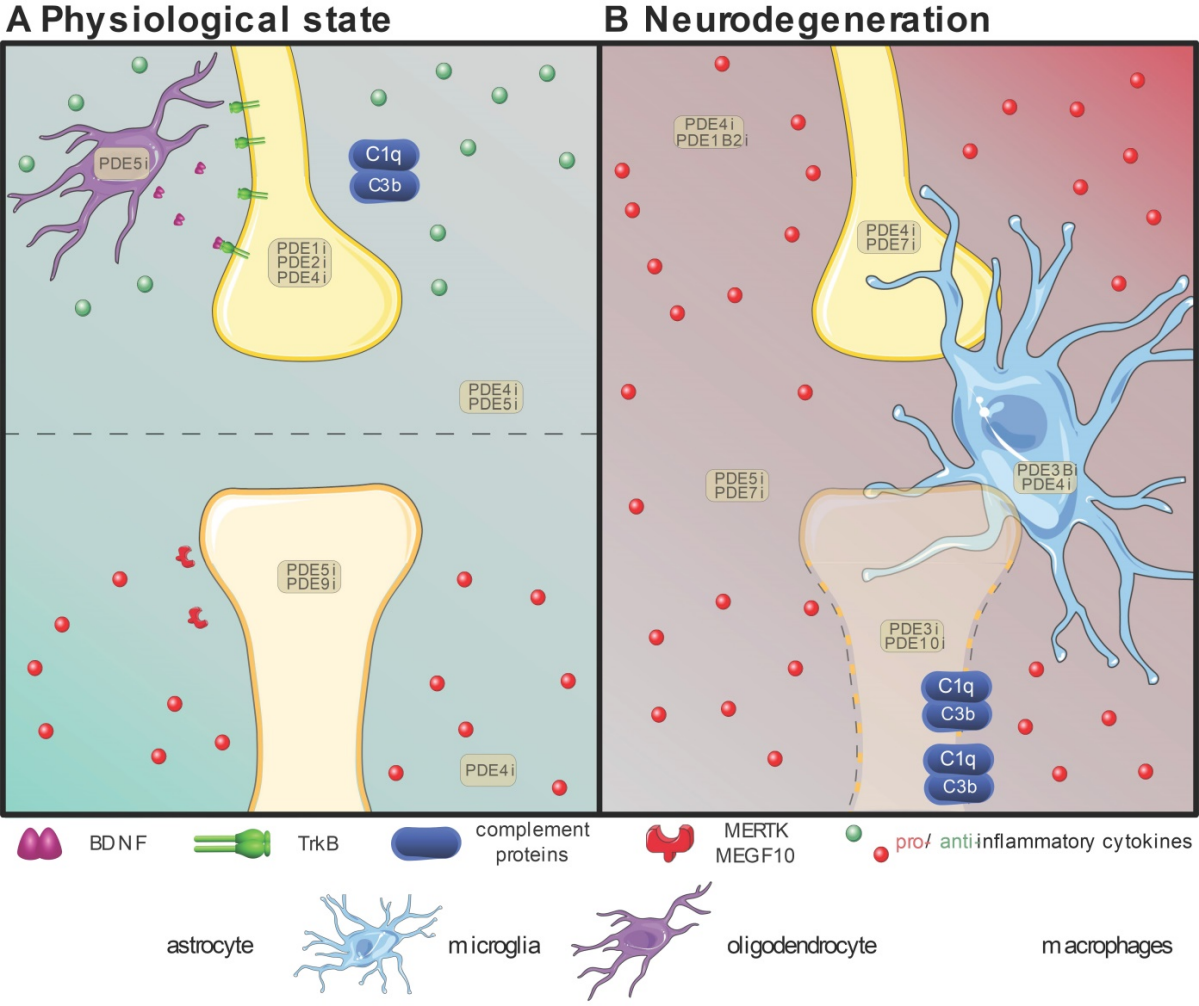
Overview of the proven effective PDE inhibition on different cell types involved in synapse maintenance or loss in the physiological state (A) or neurodegeneration (B). Resident neuroglial cells have dual roles in the physiological state (A), highlighted by the presence of pro-(red) inflammatory and anti-(green) inflammatory cytokines. The positive effects on individual synapses are shown above the dotted line, while detrimental effects are shown below. In neurodegenerative pathologies (B), in which disruption of the blood-brain-barrier is common, the resident neuroglia become activated, and patrolling macrophages (pale green) extravasate into the central nervous system and intermingle with microglia (bright blue). Local upregulation of complement proteins (C1q and C3b) leads to targeted clearance of synapses by microglia and astrocytes (bright green). Inhibition of various PDEs (PDEXi) has been found to influence synaptic plasticity directly in neurons and indirectly via glial cells by skewing them toward an anti-inflammatory phenotype and promoting transcription factors to express for instance Brain-Derived Neurotrophic Factor (BDNF) from oligodendrocytes (purple). Images were modified from Reactome icon library and Servier Medical Art (http://smart.servier.com/), licensed under a Creative Common Attribution 3.0 Generic License 226.

The importance of glial cells in synaptic elimination has already been established 31. Although the formation of synapses and strength of these connections is mainly attributable to neurons, the elimination of synapses that are too weak or in excess requires the involvement of glia. Mounting evidence underlines the importance of microglia 32, 33 and astrocytes 34 in synaptic elimination, highlighting neuron-glia signaling pathways crucial for synapse physiology. Astrocytes and microglia mediate this elimination by direct phagocytosis of synapses, as explained in detail below. Apart from clearing dead cells and debris, these glial cells thus actively participate in eliminating synapses 35. As many neurodegenerative disorders include an inflammatory environment, the effects of inflammation on synaptic plasticity are also evaluated below. Based on the intricate cellular collaboration at the basis of synaptic plasticity, and the prominent role of PDE signaling therein 36, 37, the aim of our review is to highlight the present understanding of the beneficial effects of PDE inhibition. Additionally, deeper insights into PDE biology and the importance of isoforms were provided, emphasizing their potential as targets for personalized medicine in different neuropathologies that feature synapse loss. The present review summarizes the known effects of PDE inhibition on cellular mechanisms contributing to the elimination of synapses in the normal and pathological brain, throughout development and during aging (shown in **Table 2 and Figure 1**) 38.

### Modulating the synaptic unit at the core of synaptic plasticity in health and disease

Loss of spines and synapse elimination is critical both in the primary and secondary phase of human brain circuit formation, *i.e.* from early development up to childhood and during adolescence. Synaptic elimination during development is well-documented, as observed in the developing neuromuscular junction in rodents. Aside from the neuromuscular junction, experiments have highlighted that synapse elimination also occurs in sensory and executive circuits of the brain. As such, synaptic elimination represents a key developmental process in the cerebellum, cortex, thalamus, and the hippocampus 30, 39, 40.

As synapse elimination occurs in different stages of life, an important distinction has to be made between normal and pathological aging. Medical advances and public health efforts have led to a substantial increase in the percentage of older adults in the general population. Aging remains an underlying risk factor for many neurodegenerative diseases and is often accompanied by notably deteriorating cognitive capacities 41. Physiological aging features neuroanatomical changes independent of pathological processes, such as a decrease in grey matter volume. This grey matter loss is not attributable to neuronal loss but rather the result of a gradual decline of dendritic arborization and synapse density. The main regions affected are the prefrontal cortex, medial temporal lobe, and hippocampus, associating with cognitive decline 42, 43. In addition, a decline in energy utilization coincides with physiological neurodegeneration 44. Loss of energy is attributable to the aging brain's hypometabolism, caused by hypoperfusion and loss of blood-brain barrier (BBB) integrity 45. Notably, excitatory synapses have relatively high rates of energy consumption, rendering them vulnerable to diminished production of adenosine triphosphate (ATP) by neurons 46. Apart from supporting neurons in maintaining baseline synaptic plasticity, glial cells also affect synaptic spine density. In these specialized signaling cascades, PDEs represent interesting players that control cellular and subcellular cAMP pools.

An important challenge of pharmacological inhibition of PDEs is the ability to translate preclinical work to therapeutics in humans. As illustrated below to a greater extent, the proven beneficial effects of PDE inhibition have been established in *in vitro* experiments or in *in vivo* animal model studies. However, to date, few PDE inhibitors have bridged the translational gap to become an approved therapy in the area of neurological disorders. Alzheimer's research, specifically, has looked into the potential of PDE inhibitors quite extensively 47, 48. The effect of PDE inhibition on different CNS cell types is discussed below and summarized in table 2. These studies largely reach the same conclusion: blocking of a PDE protein derived from a gene does not result in the desired effects. For added value without unwanted side effects, the role of the different isoforms needs to be better understood. In addition, an in-depth analysis of the importance of the distinct isoforms in the different cell types involved in neurodegeneration is needed. This review aims to highlight and summarize decades of PDE inhibition research to argue for the development of isoform-specific PDE inhibitors.

### Neurons - at the core of synaptic plasticity

The brain circuitry is known to undergo many neuronal changes throughout the human lifespan 46, 49. On the micro-level, alterations include synaptic plasticity 50. Neural activity alters the morphology and associated function of synapses 51. The creation, modification, maintenance, or elimination of synapses results in the ever-changing brain environment to facilitate signal propagation and storage of information. Electrophysiological properties, mainly long-term potentiation (LTP) and long-term depression (LTD), are at the basis of these alterations 51. Despite producing functionally opposite effects on synapse physiology, under physiological conditions, both LTP and LTD promote brain plasticity by changing synaptic strength. They can take place at the same synapse through subtle differences in NMDA receptor signaling. On the one hand, intense or persistent NMDA receptor activation can ultimately increase the presence of postsynaptic AMPA receptors, facilitating LTP. LTD, on the other hand, is induced by no or weak NMDA receptor activation, among other ways. The occurrence of LTD in the adult brain reduces synaptic strength, leading to the removal of postsynaptic AMPA receptors and, ultimately, when persistent, loss of spines 25, 30, 52.

Synapses are subject to activity-dependent competition. Active synaptic connections are retained, while poorly used synapses are prone to elimination. Furthermore, coincident activity of pre- and postsynaptic compartments leads to synapse strengthening. This dynamic nature of synapse strengthening or elimination enables a high degree of plasticity, which is crucial in learning and memory processes. Accordingly, synapse elimination would promote plasticity as long as it is appropriately targeted to rather inactive synapses. In neurodegenerative diseases, a decline in plasticity can be observed due to deficient strengthening and excessive elimination of synapses, even for adequately used synapses. Importantly, the increased inflammatory state in the aging and neurodegenerative brain, mediated by overactive astrocytes, microglia, and macrophages, poses a challenge for the maintenance and strengthening of these synapses. As such, excessive and non-specific synapse loss in neurodegeneration can be limited by promoting synaptic strengthening and dampening inflammatory signaling.

Activity-dependent synapse strengthening is tightly regulated by and dependent on cyclic nucleotide signaling cascades. Upon receptor activation on the postsynaptic neuron, calcium influx and G-protein coupled receptor stimulation promotes the synthesis of cGMP and cAMP. The second messengers cGMP and cAMP can then, either directly or via downstream effectors, modulate both transient and prolonged plasticity changes 5. Transient plasticity changes consist of, for example, phosphorylation of AMPA receptors, inducing trafficking into the postsynaptic membrane 4. Retrograde nitric oxide (NO) signaling enhances cGMP signaling presynaptically, a phenomenon that can promote presynaptic neurotransmitter release. More sustained plasticity effects involve mRNA translation and *de novo* protein synthesis (*e.g.*, via cAMP response element-binding protein (CREB)-mediated transcription 53) that produce more reliable postsynaptic densities (*e.g.*, by increases in scaffolding proteins such as PSD95 50), synthesis of more receptive synapses (*e.g.*, owing to more membrane-bound AMPA receptors 4), or increased paracrine and autocrine signaling to stimulate neuronal survival (*e.g.*, via brain-derived neurotrophic factor (BDNF) 54). Pharmacological PDE2 inhibition increased hippocampal LTP, neuronal plasticity, and BDNF levels both in healthy animals and in disease models 55-57. PDE3 inhibition showed restorative effects upon neuronal loss in the hippocampus through stimulation of CREB-mediated proliferation 58, while neurons were also protected by PDE10 inhibition in the context of Huntington's disease 59. Modulation of neuronal cGMP specifically can be achieved by inhibition of mainly PDE5 and PDE9. Enhanced neuronal survival through BDNF and CREB signaling and increased membrane-bound AMPA receptors was observed upon PDE5 inhibition in Alzheimer's models 4, 60, 61. Furthermore, inhibiting PDE4 and its subtypes and isoforms specifically increased CREB-mediated transcription and BDNF signaling to enhance neuroplasticity 62-64. Additionally, it has been found that ablation of the activity of the PDE4B isoform, PDE4B1, increased hippocampal CREB phosphorylation, and LTP 65.

In addition to the modulation of neuroplasticity effects, cyclic nucleotides also affect neuroinflammatory processes 14, 66. Through compartmentalization of signaling cascades, generic cAMP and cGMP can selectively influence different processes. This compartmentalization is partly established by the wide variety of PDE enzymes, enabling inhibition of certain PDEs to control inflammatory responses in neurons selectively. As cAMP and cGMP are exclusively degraded by these compartmentalized PDEs, PDEs are attractive pharmacological targets to, on the one hand, enhance neuronal plasticity and, on the other hand, reduce inflammatory responses. Moreover, in aging and neurodegenerative diseases, baseline cyclic nucleotide signaling is found to be altered, as extensively reviewed by Kelly *et al.* 3. Accordingly, inhibition of several PDE families has proven useful to enhance both transient and prolonged plasticity and dampen inflammatory responses upon insults. Levels of both cAMP and cGMP can be elevated simultaneously via inhibition of dual-specific PDEs, leading to proven beneficial effects. For example, neuronal damage by oxidative stress was minimized by the PDE1 inhibitor vinpocetine 67. In addition to PDE9 inhibition counteracting the oxidative stress and reduced plasticity in Alzheimer's disease 68, cAMP-elevating PDE4 inhibition was found to reduce neurotoxicity induced by amyloid-β 12. Similarly, the effect of lipopolysacharide (LPS)-induced neuronal inflammatory responses on neurons was diminished by the PDE4D-sparing inhibitor ABI-4 13. PDE4 inhibition also enhanced neuronal resilience and restorative capacity in different disease models 69-75. More specifically, PDE4B2 seems to be involved in mediating neuronal inflammation, as its inhibition decreased Aβ-induced TNFα and IL-1β levels 76. Given the dual therapeutic action (*i.e.*, enhancing plasticity and reducing inflammation) of PDE4 inhibition, this therapeutic strategy has been investigated in a variety of neurodegenerative disorders to protect neurons and their functional synapses 16. Despite the therapeutic potential of PDE4 inhibitors, clinical use has been plagued by the occurrence of adverse side effects (*e.g.*, headaches, diarrhea, and nausea) 77. Consequently, more selective inhibition of PDE4 subtypes or isoforms is warranted. Alternatively, cAMP signaling can be elevated by other cAMP-selective PDEs to circumvent PDE4-mediated side effects; PDE7 inhibition also displayed anti-inflammatory and neuroprotective effects 78, 79. Although PDE8 is expressed in the brain, and several PDE8 inhibitors do exist, its role in neuronal plasticity has not yet been investigated 80, 81.

cAMP compartmentalization plays a vital role in synaptic plasticity. The AKAP5-PDE4D5 complex, which is attached to the postsynaptic membrane, regulates cAMP signaling and plays a crucial role in learning and memory by facilitating activation of AMPA receptors in response to a signal 5. *In vitro* and *in vivo* studies demonstrated that LRRK2 is responsible for the localization of cAMP/PKA signaling in dendritic spines and negatively regulates cAMP/PKA signaling during synaptogenesis. In particular, LRRK2 knock-out mice exhibit increased PKA phosphorylation of AMPA receptors, leading to abnormal dendrite morphology. Thus, LRRK2/cAMP complexes play a dual role, protecting neuronal cells from inflammation and balancing intracellular signaling for the healthy development of dendrites during synaptogenesis. Furthermore, research has shown that LRRK2 is associated with Parkinson's disease 82. Yotiao, a specific splice variant of AKAP9, is found in neurons and neuromuscular junctions. This protein binds PKA and NMDA receptors, localizing cAMP signaling close to the receptor and modulating calcium permeability of the receptor 83. Although Yotiao has not been shown to bind to PDEs, its longer form, AKAP9, anchors PDE4D3, restricting cAMP signaling in close vicinity to the centrosome, and participates in microtubule dynamics 84. Two AKAP9 variants have been associated with AD; however, their contribution to neurodegeneration via cAMP/PKA signaling remains to be explored 85.

Ample research has detailed the active role of neurons in phagocytic signaling. For instance, neurophagy, the phagocytosis of live neurons and synapses by glia, is dependent on active intercellular signaling. The neuronal cell surface displays various “eat-me” signals to attract and activate phagocytic glia 86. Conversely, TAR DNA Binding Protein 43 kDa (TDP43) and CD47 are surface molecules expressed by neurons to avoid phagocytic clearance, thus called “don't eat me” signals 87, 88. Expression of CD47, in particular, is regulated by transcription factors signal transducer and activator of transcription 3 (STAT3) and nuclear factor κB (NF-κB). Consequently, these downstream effectors of cAMP signaling can benefit from PDE inhibition.

### Microglia - the brain's residing overseers

Microglia develop from erythromyeloid progenitor cells. During early development, they travel via the bloodstream and infiltrate the brain 89. Once they arrive, microglia populate the brain by proliferation and migration (reviewed by Smolders *et al.* 90). Thereafter, the influx of circulating bone marrow-derived progenitors is redundant, as the self-renewal capacity of brain-resident microglia accounts for their maintenance and local expansion in the healthy adult brain 91. As the resident macrophage population, microglia act as the first and foremost form of active innate immune defense in the brain. Micro-organisms, damaged cells, neurofibrillary tangles, or plaques activate microglia and consequently strengthen their capacity to proliferate, migrate, induce cell death, phagocytose, and present antigens 92.

Microglia also regulate the inflammatory response by producing cytokines, such as tumor necrosis factor alpha (TNF-α), interleukin-1 beta (IL-1β), chemokines, free radicals, and nitric oxide (NO) 93. Pharmacological modulation of microglial activation and synaptic elimination has garnered considerable attention as a therapeutic approach to treat neurodegenerative diseases. Elevating microglial cAMP and cGMP levels has been shown to counteract inflammatory activation and promote neuroprotection. Inhibition of dual substrate PDE3, cAMP-specific PDE4 and cGMP-specific PDE5 using amrinone, rolipram, and sildenafil, respectively, drives cultured microglia towards an anti-inflammatory phenotype with decreased production of TNF-α, IL-1β, IL-12 and NO, suggesting a neuroprotective effect exerted via the microglial cGMP or cAMP signaling pathways 94-96. The non-selective PDE inhibitor pentoxifylline decreases LPS-dependent increases in TNF-α 97.

PDE4 inhibitors (including rolipram) and a PDE4D-negative allosteric modulator (D158681) 98 have pro-cognitive, neuroprotective, and anti-inflammatory effects *in vivo* (reviewed in 99). Of the PDE4 subtypes that are known to modulate inflammation in the CNS, the high expression of PDE4B in microglia seems to underline its importance. Notably, PDE4B is highly expressed in activated microglia found in traumatic brain and spinal cord injury 99. However, inhibition of PDE4 subtype expression is not microglia-specific; its effects cannot be directly attributed to microglia only in *in vivo* studies. Microglial cAMP and cGMP have also been shown to upregulate Arginase (*Arg-*1) in microglia, while sildenafil has been found to upregulate chitinase 3-like 3 (YM-1), skewing them towards an anti-inflammatory phenotype 96, 100. In inflammatory reactions, Toll-like receptors (TLRs) amplify the release of pro-inflammatory cytokines and chemokines. The non-selective PDE inhibitor Ibudilast, targeting PDE4, -10 and to a lesser extent, PDE3 and -11, has been shown to antagonize TLR4 101. Interestingly, cAMP/PKA signaling is anchored and regulated by the leucine-rich repeat kinase 2 (LRRK2)-PDE4 complex. Although LRRK2 is not microglia-specific, research has shown that its knock-out decreased the LPS-dependent pro-inflammatory response of microglia by modulation of cAMP/PKA activity *in vivo* 102. It has also been shown that LRRK2 restricts cAMP signaling mainly to intercellular endosomal-lysosomal compartments and mitochondria, affecting vesicle trafficking and autophagy 103. These findings support the fact that PDEs associated with cAMP/AKAP complexes and their localization have to be taken into account when designing drugs to improve neuroprotection and synaptogenesis.

In addition to their protective functions, microglia are critical cells in overall brain maintenance. Both during development and homeostasis, microglia actively survey and shape the brain parenchyma by physically monitoring neurons and synapses 49, 104, 105. This contact is dependent on neuronal activity: the frequency of these contacts decreases with decreasing neuronal activity and the duration of contact increases in pathological circumstances 106, 107. During development, an excess of newly born neurons is generated, and microglia quickly clear dead and dying cells through phagocytosis to prevent the spillover of pro-inflammatory and neurotoxic molecules 108-110. Similarly, microglia are vital regulators of the balance between synaptogenesis and synaptic elimination: synaptic connections between neurons are formed in abundance during postnatal brain development, and microglia eliminate weak synaptic connections and strengthen the remaining synapses 111. *In vivo* pre- and postnatal microglial depletion or perturbation models reveals the essential role for microglia in synapse formation, refinement, and elimination (16, 24, 62-74). Microglial cAMP and cGMP have also been shown to be critical for microglial surveillance of the brain parenchyma and, therefore, rapid and subtle manipulation of cAMP and cGMP levels can dramatically affect their role in synaptic elimination. The addition of the non-selective, also called pan-PDE blocker, IBMX, prevents the breakdown of cAMP and triggers a fast retraction of microglial processes *in situ*, leading to reduced elimination. Specific blocking of PDE3B, whose expression is highly enriched in microglia, has a similar effect, suggesting that intracellular cAMP and cGMP levels regulate the morphology and sensing mechanism of microglia 112. General microglial phagocytosis is also regulated through cAMP and cGMP signaling. In addition, PDE4B inhibition has been shown to alter microglial phagocytosis and reactivity, as inflammation regulates PDE4 expression (reviewed in 113). Treating microglia with IBMX (all PDEs), rolipram (PDE4), or cilostamide (PDE3) resulted in inhibition of microglial phagocytosis 114, suggesting a potential mechanism to counteract phagocytosing of synapses 115. It is clear that the structural and functional consequences of impaired microglial surveillance of the neuronal network require further investigation.

An important point to raise is that *ex vivo* live-imaging has not found evidence of active phagocytosis of complete synapses by microglia 116. The same study did confirm direct engulfment and subsequent elimination of pre-synaptic material. The distinction is that active phagocytosis (which was not witnessed) entails direct phagocytosis of spines as a functional unit on neurons, constituting direct breakdown of functional spines by microglia. Conversely, elimination of synaptic material encompasses the clearance of synaptic debris following disruption of the synapse or targeting of the pre-synaptic compartment. More specifically, the authors observed trogocytosis -or nibbling- of presynaptic boutons, while contacts at postsynaptic sites were shown to elicit transient filopodia from mature spines. A microglia depletion study suggests that microglial support in synapse formation is maintained throughout the lifespan, while microglial-mediated synapse elimination declines from adolescence 117. However, synaptic elimination might be turned back on aberrantly in the aged and diseased brain 118.

Synaptic elimination involves many cues that promote and inhibit the detection and removal of specific synapses (reviewed in 119-121). To date, the best-described mediator of synaptic elimination is the classic complement cascade. The soluble complement protein C1q targets synapses that need to be eliminated in an activity-dependent and transforming growth factor beta (TGF-β)-induced manner, after which a sequence of proteolytic reactions leads to the covalent association of C3b proteins to the same synapses. The complement receptor CR3 (alternatively CD11b/CD18), which is expressed on the surface of microglia, binds C3b and promotes phagocytosis of the opsonized synapses 122. The importance of functional cell-cell communication is again highlighted in this process. TGF-β is produced by astrocytes, and its binding to its specific receptor on neurons triggers these neurons to produce C1q, which functions as an 'eat me' signal for microglia 123. Conversely, TGF-β is needed by neurons for conversion from early phase LTP to late phase LTP and correlates with an increased expression of the phosphorylated transcription factor CREB, downstream of cAMP signaling 124.

The classic complement cascade ensures the removal of overproduced synapses during postnatal development, and the expression of its components decreases in adulthood 125. Tight regulation is a necessity, as dysfunctional synaptic elimination leads to different neuropathologies depending on age and the affected brain region. Complement-dependent synaptic elimination is implicated in, among others, AD 118, frontotemporal lobar dementia 126, West Nile virus-associated neurodegeneration 127, schizophrenia 128, 129, epilepsy 130, and multiple sclerosis 131. In contrast, microglia and the complement system were initially seen as neuroprotective in AD, as the complement system counteracts amyloid plaque deposition by inducing microglial phagocytosis of Aβ 132. In a later stage, by encapsulating and thereby segregating the amyloid plaques, microglia appear neuroprotective. Thus, microglial functioning has been extensively described as being a double-edged sword in several neuropathological disorders. However, studies investigating microglial ablation found no difference in plaque burden, but showed considerable cognitive improvement, which suggests an alternate, neurodegenerative process mediated by microglia in AD 29, 133. All in all, disturbances in the complement cascade may represent a shared etiological pathway in neurological diseases that needs to be further explored.

Age-dependent synapse loss is characterized by a decline in cognitive functions and is provoked by a combination of increased synaptic elimination, loss of cortico-cortical connections, and neuronal apoptosis 134. Aging mouse and human brains show increased levels of components of the complement system, such as C1q 135. During adulthood and in normal aging, microglia produce the majority of C1q, which is a harbinger for increased synaptic elimination 136. Aging microglia also upregulate the expression of CR3, which implicates a microglial contribution in synaptic loss during normal aging that utilizes the complement pathway 137. Increased expression and presence of CR3 is one of the possible mechanisms explaining phagocytic clearance of focal apoptotic synaptic compartments by microglia, the other being increased Ca^2+^ levels in dendritic spines, triggering 'eat me' signals for microglia 138. Aging microglia have also been shown to be activated, accompanied by elevated levels of chemokines and cytokines such as TNF-α and IL-1β 139, and an altered expression of various membrane receptors like triggering receptor expressed on myeloid cells 2 (TREM2), scavenger receptor class A (SR-A/Scara-1), and CD33 140. This increased activation state can result in excessive synaptic elimination. Interestingly, many pathways found in studies aimed at elucidating the mechanisms behind microglial phagocytosis share cAMP as an upstream effector 141, 142. Administering PDE inhibitors, like IBMX and rolipram, or reagents that alter cAMP levels has been shown to inhibit phagocytosis in macrophages 115, but also phagocytosis of myelin mediated by CR3 and SR-A 141. The mechanisms underlying age-dependent changes in microglial function remain elusive, but are thought to be similar, at least in part, to those in neurodegenerative diseases.

### Macrophages - the reinforcements for hire

The central myeloid cell population of the CNS is composed of parenchymal microglia and non-parenchymal macrophages, including perivascular macrophages, meningeal macrophages, macrophages of the choroid plexus and blood-borne monocytes 143-145. Non-parenchymal macrophages in the CNS are known to be crucial for the preservation of capillary stability and BBB integrity 146. In the past few years, CNS macrophages have gained interest for their involvement in synaptic maintenance and associated CNS disorders. Even though the process of synapse refinement has been mainly attributed to microglia, it is likely that non-parenchymal macrophages also interact with neurons, as perivascular and meningeal macrophages even show increased phagocytic activity when compared to microglia 147. Blood-borne monocytes, once in the brain, polarize into monocyte-derived macrophages, which subsequently share similar characteristics with parenchymal microglia (*e.g.*, the expression of complement receptors, signal regulatory protein alpha (SIRPα), chemokine receptors, and TREM2) 148, 149. Studies using *in vitro* co-cultures of monocyte-derived macrophages and neurons have further emphasized the possible involvement of these macrophages with regards to synapse refinements, as synapse material was found present within these macrophages 148, 149.

During aging, blood-borne monocytes infiltrate the brain parenchyma, where they differentiate into microglia-like monocyte-derived macrophages 148. The monocyte-derived macrophage population subsequently becomes intermingled with tissue-resident microglia and either facilitates or counteracts neurological signaling 143, 145. In the aged brain, peripheral monocytes infiltrate to a greater degree and consequently alter the CNS microenvironment, skewing it to a more inflammatory environment. The infiltrated monocytes negatively influence synaptic plasticity, as they decrease the LTP response in hippocampal brain slices, suggestive of a detrimental effect of infiltrated macrophages 150, 151. *In vivo* studies further confirmed the destructive nature of the infiltrated macrophages, since the level of macrophage infiltration in aged rats correlated with reduced synaptic functioning 152. This age-related pro-inflammatory environment of the CNS has negative consequences. Research has shown that aged animals are less protected against acute traumatic CNS insults, as they exhibited an increase in macrophage infiltration leading to exacerbated synapse loss and accelerated cognitive decline 153. Rolipram-mediated PDE4 inhibition increased the migratory capacity of a murine macrophage cell line when challenged with LPS, an outcome that could lead to a more efficient pathogen clearance and subsequent wound repair 154. This migratory phenotype was primarily mediated by activating the cAMP-Epac signaling cascade 154.

Even though the exact role of non-parenchymal macrophages remains inconclusive, several current studies have aimed at skewing specific macrophage-mediated responses to resolve neurodegenerative disease. One strategy to regulate macrophage functions is to alter intracellular levels of second messengers by inhibiting specific PDEs. To understand which type of PDE inhibition could be beneficial in particular processes, differences in PDE expression profiles have been mapped. Interestingly, when peripheral monocytes differentiate towards macrophages *in vitro*, dramatic changes can be observed with regards to PDE expression 155. For instance, while monocytes are characterized by profound PDE4 activity, its activity rapidly declines during differentiation 155. A detailed PDE4 isoform profiling showed a selective downregulation of PDE4D3 and PDE4D5. Conversely, PDE4A10 and PDE4B2 protein levels increased, shifting the primary source of PDE4 activity from long PDE4D isoforms in monocytes to short PDE4B isoforms in macrophages 156. While the total PDE4 activity reduced during monocyte to macrophage differentiation, PDE1 and PDE3 activity increased 155.

The importance of synaptic maintenance and preservation is especially highlighted in schizophrenia. In schizophrenic patients, an increase in the absolute number of circulating peripheral blood monocytes was found, whereas an increase of macrophages was observed in the cerebrospinal fluid of schizophrenia patients during an acute psychotic episode 157, 158. However, when differentiating peripheral monocytes from schizophrenia patients or healthy controls into macrophages *in vitro*, only a minor decrease in purinergic receptor P2X7 gene expression was seen in schizophrenia material upon pro-inflammatory LPS stimulation 159. No other functional irregularities were present in response to alternative pro- or anti-inflammatory stimuli, questioning the active involvement of non-parenchymal macrophages in alleviating or exacerbating schizophrenic disease progression.

Owing to their phagocytic capacity, macrophages have been extensively investigated for their possible neuroprotective potential in AD, as they may regulate Aβ clearance 160. In both AD patients and animal models for AD, macrophages have been shown to accumulate around senile plaques 161, 162. When stimulating perivascular macrophage turnover, a reduced cerebral amyloid angiopathy load was observed in TgCRND8 mice, an outcome that was found to occur independent of microglia-mediated Aβ clearance 163. The role of not only perivascular but also monocyte-derived macrophages has been studied in relationship to AD. Both *in vitro* and *in vivo* studies have showed an increased capacity of these monocyte-derived macrophages to remove Aβ fibrils, a process mediated by intracellular CD36/early endosomal antigen-1 (EEA1) early endosomal proteolysis 164-166. Interestingly, not only fibrils, but also Aβ42 oligomers, were shown to be cleared by activated macrophages *in vivo* 166. In contrast to fibrils, oligomer clearance was primarily mediated via extracellular/matrix metallopeptidase 9 (MMP-9) enzymatic degradation, leading to macrophage-mediated vesicular glutamate transporter 1/postsynaptic density protein 95 (VGlut1/PSD95) synapse preservation through the prevention of oligomer build-up and subsequent synaptotoxicity 166.

Understanding isoform-specific PDE expression changes can offer insights into PDE inhibitor development as a therapeutic strategy to alter macrophage-mediated responses in the CNS. As such, PDE inhibitors can be beneficial in multiple CNS disorders, either by changing the phagocytic capacity of macrophages or by downregulating their pro-inflammatory markers and therefore resolving neuroinflammation. Besides PDE4, PDE1B2 suppression has been studied for its effect on macrophages. When PDE1B2 was specifically suppressed, macrophages showed an increased phagocytic ability and enhanced cell spreading 167. Finally, the pro-inflammatory microenvironment in the aged and diseased brain has been shown to increase PDE4B2 expression in macrophages, specifically (138). Therefore, inhibiting the PDE4B family or specific isoforms is an interesting strategy for controlling the age-related pro-inflammatory environment in the brain.

There is little information available about the compartmentalization of second messengers within CNS macrophages. Evidence has been found to show that AKAP10 (AKAP-D) and AKAP95 regulate secretion of anti-inflammatory cytokines IL-10, IL-6, and TNF-α through cAMP/PKA signaling in alveolar macrophages 168. AKAP95, observed in nuclear fractions, formed complexes with nuclear PDE4 and PDE3, where the latter serves as a barrier to prevent the accumulation of cAMP 169.

### Astrocytes - from support structure to clean-up crew

Astrocytes are the most abundant cells within the CNS. Their supportive and protective role renders them indispensable in normal brain functioning. As such, astrocytes are involved in extracellular matrix (ECM) homeostasis, neurotransmitter recycling, neuroplasticity, and many other processes. Their particular contribution to synaptic formation, maintenance, and elimination has become more appreciated over the past decades. Astrocytes secrete synaptogenic factors, such as thrombospondins, which are required during synaptic formation and act to strengthen functional synapses 170. Furthermore, through direct physical contact with astrocytes, synaptogenesis happens considerably faster (as evidenced *in vitro* 171), emphasizing the importance of these glial cells during synaptogenesis 172. As part of the so-called 'tripartite synapse', along with pre- and postsynaptic neurons, astrocytes exert a wide range of regulatory functions to maintain synaptic stability. However, here, we mainly focus on their role in synaptic elimination and E/I balance maintenance during neurodegeneration.

In addition to microglia, astrocytes have been shown to be involved in synaptic elimination. A study by Chung *et al.* showed that astrocytes express phagocytic receptors, such as MER Proto-Oncogene, Tyrosine Kinase (MERTK) and multiple epidermal growth factor like domains 10 (MEGF10), which are responsible for synapse targeting and elimination 170, 173. Interestingly, the phagocytosis of synapses by astrocytes seemed to be independent of the complement protein C1q, suggesting a different phagocytic pathway than employed by microglia. Additionally, the study revealed that the phagocytic functions of astrocytes are strongly dependent on neural activity, indicating that astrocytes actively prune active synapses rather than just clean up synaptic debris. Specifically, blocking spontaneous retinal waves in both eyes significantly reduced astrocyte-mediated phagocytosis of bilateral synaptic inputs, whereas selective blocking of activity in only one eye induced preferential engulfment of the silenced synapses by astrocytes 173. Reactive astrocytes have also been shown to ?? phagocytose synapses in hippocampal regions of amyloid precursor protein/presenilin 1 (APP/PS1) mice as well as in post-mortem AD brains. Dystrophic presynaptic vGlut1-positive terminals seem to be targeted and degraded by astrocytic endfeet, yet it remains unclear whether these mechanisms contribute to disease pathology or become impaired during disease progression 174. The involvement of PDEs in astrocytic-mediated synaptic control remains an unstudied topic. PDE inhibition or cAMP elevation within astrocytes seems to regulate distinct neuroinflammatory pathways and could, therefore, influence synaptic integrity in an indirect manner 66. Treatment of astrocytes with 8-Br-cAMP, a cAMP analog, has been shown to decrease the levels of the pro-inflammatory cytokine TNFα 11. TNFα stimulates C3 expression, facilitating complement-mediated synapse elimination, suggesting that astrocytic cAMP might attenuate inflammatory induced synaptic elimination 175, 176.

Reactive astrocytes have also been shown to be closely associated with amyloid plaques in AD brains and were found to internalize Aβ both *in vitro* and *in vivo* 177-179. Subsequent astrocytic Aβ-induced release of pro-inflammatory factors leads to synaptic disturbances and neuronal death in models for AD 180, 181. A genotype-specific phagocytic ability of astrocytes has been shown within the context of AD. Astrocytes of apolipoprotein E 4 (APOE4) knock-in (KI) mice exhibit a defective phagocytic capacity, while astrocytes from APOE2 KI mice have an enhanced rate of synaptic elimination 182. Interestingly, induced pluripotent stem cell-derived astrocytes from APOE4 AD patients that had been genetically converted with CRISPR-Cas9 to an APOE3 genotype showed a rescued overall phagocytic capacity, confirming the APOE allele-dependent phagocytic capacity of astrocytes in AD pathology 183.

Astrocytes target both excitatory and inhibitory synapses, yet the balance varies during different developmental stages. In the first phase of postnatal development, more excitatory synapses are engulfed, likely owing to the higher rate of synaptogenesis in this phase. This observation shows that astrocytes play a pivotal role in maintaining the E/I equilibrium 173. One of the significant functions of astrocytes in the vicinity of excitatory synapses is the reuptake of excessive glutamate via the excitatory amino acid transporters (EAAT)1, and EAAT3, to prevent excitotoxicity 184. Both amyotrophic lateral sclerosis (ALS) and Huntington mutant mouse models, *i.e.*, superoxide dismutase 1 (SOD1)- and huntingtin (htt)-mediated, respectively, show defective astrocytic glutamate transporters, eventually leading to extracellular glutamate accumulation and neuronal death through excitotoxicity 185, 186. Similarly disrupted glutamate receptors have been proposed within the context of Parkinson's disease (PD), resulting from α-synuclein accumulation within astrocytes 187. Furthermore, astrocytes are capable of producing and releasing GABA as a gliotransmitter, potentially activating GABA receptors on adjacent neurons 188. However, reactive astrocytes produce markedly less glutamine. As a precursor for GABA, glutamine starvation leads to a reduction in inhibitory currents as a result of GABA depletion, while excitatory currents remain unaltered 189. In contrast, overreactive astrocytes in AD have been shown to disrupt synaptic function through excessive production and release of GABA by the GABA-producing enzyme monoamine oxidase B (MAO-B). The released GABA strongly inhibits neuronal synaptic release, leading to impairment in LTP and learning and memory 190. In line with this, it has been shown that Aβ increases astrocytic GABA synthesis, suggesting a crucial role of astrocytes in the E/I imbalance in AD 191. The opposite has been shown in a mouse model for Huntington's disease (HD), where astrocytic GABA release through GABA transporter 3 (GAT-3) is reduced, resulting in reduced tonic inhibition in striatal output neurons 192. It has been shown that PDE5 inhibition through sildenafil treatment has a preventive and restorative effect on LPS-induced inflammation in astrocytes *in vivo* 193. Accordingly, ibudilast administration has been proven to reduce astrogliosis during neuroinflammation and seems to prevent astrocytic apoptosis via cGMP signaling 15, 194, 195. Inhibition of PDE7 by the inhibitor S14 has been shown to stimulate astrocyte-mediated Aβ degradation in a murine model for AD 79. Taken together, these data highlight the therapeutic potential of PDE modulation in astrocytes within the context of neuroinflammation. However, further research is necessary to find whether PDE inhibition has a direct effect on astrocytic-mediated synapse maintenance. Since astrocytes are crucial mediators during synaptic elimination and in preserving the E/I balance, future research should focus on these mechanisms to elucidate the potential benefits of PDE inhibitors as therapeutic strategies for neurodegenerative diseases.

### Oligodendrocytes - silent modulators of the synaptic unit

Oligodendrocytes are indispensable in CNS functioning. In the case of synapse loss, however, their role is not yet fully understood. The supportive, seemingly static role of oligodendrocytes reaches well beyond their prominent role as myelinating cells of the CNS; they are active participants in cell-signaling pathways. The oligodendrocytic processes are in intimate contact with neurons at both pre- and postsynaptic structures, facilitating nutritional and structural support for synapses 100, 196. Therefore, oligodendrocyte cell signaling pathways should not be overlooked in the context of synaptogenesis. Cyclic nucleotide signaling has proven to be vital in oligodendrocyte metabolism, implicating them as interesting effector cells 66. For instance, inhibition of the cGMP-specific PDE5 by sildenafil improves BDNF signaling of oligodendrocytes and thus synaptogenesis 100. The role of compartmentalized cAMP/PKA and cGMP/PKG signaling, to the best of our knowledge, remains to be elucidated in oligodendrocyte functioning pathways.

Oligodendrocytes are the myelinating glia of the brain. Though proven to be indispensable for brain functioning, to the best of our knowledge, they have not been studied extensively in the context of synapse development, maintenance, or plasticity. As previously illustrated, glial-secreted factors are of undeniable importance for synaptic maturation and maintenance. Notably, in the developing brain, oligodendrocytes actively participate in synaptic plasticity through BDNF signaling, promoting neuronal survival and plasticity by inducing LTP 197, 198. BDNF plays a fundamental role in age-related synaptic loss, preventing cerebral atrophy and cognitive decline 199, 200. Oligodendrocyte-astrocyte crosstalk has been proven to be of importance in the formation and maintenance of the CNS. Importantly, these interactions are not only involved in activity dependent adaptive myelination but also provide metabolic support to axons 201-203.

Studies looking into the involvement of oligodendrocyte precursor cells (OPCs) have found evidence of them receiving signals from axons for myelination in an activity-dependent manner via glutamatergic signaling. The abundantly present AMPA- and NMDA-type glutamate receptors mediate OPC proliferation and migration or myelination, respectively 204, 205. An essential functional overlap in synaptic strengthening and myelination by oligodendrocytes has been noted in the process of learning. Both processes are enhanced in animals living in an enriched environment, prompting the conclusion that oligodendrocytes are involved in cell-cell communication processes that take place during learning. Proliferation and differentiation of OPCs have been shown in animals subjected to an enriched environment that involves learning exercises 206, 207. As PDE4 inhibition, mainly by rolipram 208, 209, has proven to significantly enhance OPC differentiation, this process may aid in creating a positive environment for synaptic plasticity. OPCs receive synaptic input from pyramidal neurons and interneurons in the hippocampus and axons in the corpus callosum 210. How exactly this communication between neurons and oligodendrocytes takes place remains to be elucidated, and understanding this process should contribute to efforts to counteract synapse loss. In addition, cAMP and PDE remain important to oligodendrocyte signaling. By modulating these signaling mechanisms, and skewing them towards a differentiated state, trophic support can be used as a driving factor in synaptic plasticity. The associated activity-induced myelination is a byproduct of PDE inhibition, which contributes to efficient support of the neuronal network.

## Concluding remarks

PDEs have been at the center of many investigative strategies aimed at understanding various diseases. Increased understanding of signaling cascades surrounding specific PDE isoforms has uncovered their potential as therapeutic targets. As non-selective inhibition often proves ineffective or is accompanied by adverse effects, only a small number of PDE inhibitors have translated to the clinic, and many inhibitors still require validation for clinical neuromodulation 48, 211.

Elevation of cyclic nucleotide levels in astrocytes ameliorates neuromodulatory processes, contributing to a suitable environment for synaptic plasticity. In addition to astrocytes, microglia are the other neuroglial cells directly interacting with synapses. They influence not only elimination, but also the formation and protection of synapses. This influence is exerted during development and learning, as well as in aging and neurodegeneration. In the latter case, when neuroinflammation is widespread, PDE inhibition has proven useful in counteracting the activation of microglia, limiting the effect of inflammatory signaling. Additionally, the phagocytic capacity of astrocytes and microglia contributes to the elimination of synapses via its focal clearance. Comparison of astrocytic and microglial phagocytosis addresses an interesting knowledge gap, as it remains unclear if and, if so, how neural activity controls the rate of astrocyte-mediated synapse phagocytosis, whether astrocytes and microglia phagocytose different synapse types or circuits, and which different 'eat me signals' are involved 173. If different receptors with their respective signaling molecules are at the basis of phagocytosis by these two cell types, this fact presents an opportunity for differential PDE inhibition interventions. The inhibition of PDEs can counteract inflammatory pruning by microglia and astrocytes.

Notably, the effects of PDE inhibition have a direct modulatory effect on neurons, contributing to synaptic plasticity and neuroprotection. Both transient and lasting changes on plasticity can be achieved by targeting neurons, ultimately strengthening the synaptic network. The direct effects of the inflammatory environment on neurons can be affected by PDE inhibition. Previous work digging deeper into targeting isoforms of PDEs has focused largely on neurons, yet an exciting avenue might be to pursue glial cells. The effects of isoform compartmentalization are readily apparent in various forms of synaptic plasticity in neurons, so finding out to what extent varying isoforms alter glial cell functioning might prove valuable to understanding neurodegeneration. An interesting avenue that has been the focus of many research labs is the neuronal expression of intercellular signaling molecules that can activate surrounding glial cells. Many of these signals have second messengers lying downstream or upstream to PDE-directed pathways, rendering them vulnerable to PDE signaling 212, 213.

In closing, the role of glial cells and glia-neuron communications has gained increasing interest as a driving factor behind many diseases. Enhancement of both cAMP and cGMP signaling can successfully promote synapse strengthening and diminish inflammatory signaling, as is evident by the use of a variety of PDE inhibitors. As such, PDE inhibition has potential as a therapeutic approach to make neurons and their synapses more resilient to (disease-induced) glia-mediated elimination. The usefulness of PDE inhibitors is not limited to protecting neurons, however. By actively altering cell signaling in glial cells, a more direct approach can also be pursued. Recent advances in understanding different neuropathologies have opened up interesting avenues towards personalized medicine. As summarized in this review, the specific expression pattern of PDEs in different cell types allows targeted treatment strategies. Expanding on this notion, the targeting of isoform-specific PDE signatures per cell type may prevent neuroinflammation, while promoting synaptic plasticity in divergent pathologies. Because of this, the holistic impact of PDE inhibition can be beneficial to the entire synaptic unit. Consequently, combination therapy consisting of inhibitors targeting multiple PDEs, over multiple cell types, paves the way towards personalized medicine. This underlying potential solidifies that PDE inhibitors remain a viable strategy, yet require additional research to reach their full potential. Although the use of PDE inhibitors can lead to adverse side effects, targeting of specific isoforms holds promise to circumvent these unwanted effects. By determining the predominant isoforms of PDEs in fundamental pathways of neurodegenerative diseases, a deeper understanding of their causative mechanisms can be achieved. The additive effect of combining multiple isoform-specific drugs into pathology-specific therapy holds promise to finally unlock the full potential of PDE inhibitors.

## Figures and Tables

**Table 1:** Summarizing different PDE genes with their substrate specificity and relative expression in different murine or human CNS cell types

Gene family	Gene	Substrate	Cellular expression (murine)				Cellular expression (human)			
			Oligodendrocytes	Microglia/macrophages	Astrocytes	Neurons	Oligodendrocytes	Microglia/macrophages	Astrocytes	Neurons
PDE1	PDE1A	Dual	-*	-	-	+++	+++	-	-	+++
	PDE1B		-*	+	+++*	+++	-	+	-	+++
	PDE1C		+*	-	+	+	+++	-	+	+
PDE2	PDE2A	Dual	+*	+*	-	+++*	-	-	-	+
PDE3	PDE3A	Dual	-	-	+++	+*	-	-	+++	+++
PDE4	PDE4A	cAMP	+*	+*	+*	+++*	-	-	-	+
	PDE4B		+*	-*	+++*	-*	+++	+++	+	+
	PDE4C		-	-	-	-	-	-	-	-
	PDE4D		-	-	+++	+++				
PDE5	PDE5A	cGMP	-	-	-	-				
PDE6	PDE6A	cGMP	-	-	-	-	-	-	-	-
	PDE6B		-*	-	-	-	+	-	-	-
	PDE6C		-	-	-	-	-	-	-	-
PDE7	PDE7A	cAMP	+++*	-*	+++	+++*	+	+	+++	+
	PDE7B		-*	-	+	-*	+	-	+	+++
PDE8	PDE8A	cAMP	+++	-*	-	-*	+++	+	-	+
	PDE8B		+++*	+*	+	+++	+	-	+	+++
PDE9	PDE9A	cGMP	+*	-*	+++*	+*	-	+	+	-
PDE10	PDE10A	Dual	+	-	+	+++	+	-	+	+++
PDE11	PDE11A	Dual	-*	-	-	-	+	-	-	-

Expression per cell types is based on 214. Differences in expression between murine and human cell types pose a challenge in the context of translational capacity of PDE inhibitors. *Murine expression different from human.

**Table 2:** Overview of selective PDE inhibition per cell type

Cell type	Inhibitor	Target(s)	Specificity	Effect	*In vitro/in vivo*
Neuron	vinpocetine	PDE1	dual	minimizes neuronal damage by oxidative stress 67	*In vivo*
	Bay 60-7550	PDE2	dual	increases hippocampal LTP, neuronal plasticity, and BDNF levels 56, 215, 216	*In vitro + In vivo*
	Cilostazol	PDE3	dual	stimulates CREB-mediated proliferation in the hippocampus 58	*In vivo*
	Rolipram	PDE4	cAMP	reduces neurotoxicity induced by amyloid β 12	*In vivo*
	Roflumilast/BPN14770 Knockout/RNAi			increases CREB-mediated transcription and BDNF signaling 217-219	*In vitro + In vivo*
	Rolipram			enhances neuronal resilience and restorative capacity 70, 73, 75, 220-223	*In vitro + In vivo*
	ABI-4	PDE4A-C	cAMP	diminishes the effect of LPS-induced neuronal inflammatory responses 13	*In vitro*
	Transgenic model	PDE4B1	cAMP	increased hippocampal CREB phosphorylation and LTP 65	*In vivo*
	Ferulic acid	PDE4B2	cAMP	downregulated Aβ-induced TNFα and IL-1β levels 224	*In vitro*
	Sildenafil/vardenafil	PDE5	cGMP	enhanced neuronal survival through BDNF and CREB signaling and increased membrane-bound AMPA receptors 4, 60	*In vivo*
	BRL 50481/S14	PDE7	cAMP	displayed anti-inflammatory and neuroproductive effects 77, 78	*In vitro + In vivo*
	BAY 73-6691	PDE9	cGMP	counteracts oxidative stress and reduced plasticity 61, 68	*In vitro + In vivo*
	Transgenic model/TP10	PDE10	dual	neuroprotective effects in Huntington's model 222, 225	*In vivo*
Astrocyte	sildenafil	PDE5	cGMP	prevents LPS-induced inflammation 176	*In vivo*
	ibudilast	PDE4/10 (+3/11)	cGMP	reduces astrogliosis during neuroinflammation and prevents astrocytic apoptosis 15, 194, 195	*In vitro + In vivo*
	S14	PDE7	cAMP	stimulates Aβ degradation in a murine model for AD 79	*In vivo*
Oligodendrocyte	sildenafil	PDE5	cGMP	improves BDNF signaling 100	*In vivo*
	rolipram	PDE4	cAMP	improves OPC differentiation 208, 209	*In vitro + In vivo*
Microglia	amrinone	PDE3	dual	skewing to an anti-inflammatory phenotype with decreased production of TNF-α, IL-1β, IL-12, and NO 94, 95	*In vitro*
	rolipram	PDE4	cAMP		
	sildenafil	PDE5	cGMP		
				upregulation of YM-1 100	*In vivo*
	pentoxifylline	Non-selective	dual	decrease LPS-dependent increase in TNF-α 97	*In vitro*
	rolipram	PDE4	cAMP	precognitive, neuroprotective and anti-inflammatory effects 98, 99	*In vitro + In vivo*
	PDE4D-NAM	PDE4D	cAMP		
	ibudilast	PDE4/10 (+3/11)	multiple	antagonizing Toll-like receptor 4 101	*In vivo*
	cilostamide/amrinone	PDE3	dual	regulate morphology and sensing mechanism (filopodia) of microglia 112	*In vitro*
	IBMX	Non-selectuve	dual		
				inhibition of microglial phagocytosis 114, 141	*In vitro*
	rolipram	PDE4	cAMP		
	cilostamide	PDE3	dual		
Macrophage	shRNA	PDE1B2	dual	increased phagocytic ability and augmented cell spreading 167	*In vitro*
	rolipram	PDE4	cAMP	increased migratory capacity upon LPS challenge 154	*In vitro*

Type of inhibitor, the effect on the cell type in question, and modality of effects witnessed in the referred article is shown.

## Abbreviations

Aβ: amyloid beta
AD: Alzheimer's disease
AKAP: A-kinase-anchoring protein
ALS: amyotrophic lateral sclerosis
AMPA: α-Amino-3-hydroxy-5-methyl-4-isoxazolepropionic acid
Arg-1: arginase
APOE: apolipoprotein E
APP/PS1: amyloid precursor protein/presenilin 1
ATP: adenosine triphosphate
BBB: blood-brain-barrier
BDNF: brain derived neurotrophic factor
cAMP: 3',5'-cyclic adenosine monophosphate
CNS: central nervous system
CR: complement receptor
CREB: cAMP response element-binding protein
cGMP: 3',5'-cyclic guanosine monophosphate
CRISPR: Clustered Regularly Interspaced Short Palindromic Repeats
EAAT: excitatory amino acid transporter
ECM: extracellular matrix
EEA1+: early endosomal antigen-1
E/I: excitation/inhibition
GABA: gamma-aminobutyric acid
GAT-3: GABA transporter 3
GKAP: G-kinase anchoring protein
HD: Huntington's disease
htt: huntingtin
IBMX: 3-isobutyl-1-methylxanthine
IL1β: interleukin 1 beta
KI: knock-in
LPS: lipopolysacharide
LRRK2: leucine-rich repeat kinase 2
LTD: long term depression
LTP: long term potentiation
MAO-B: monoamine oxidase B
MEGF10: multiple epidermal growth factor like domains 10
MERTK: MER Proto-Oncogene, Tyrosine Kinase
MMP-9: matrix metallopeptidase 9
NMDA: N-methyl-D-aspartate
NO: nitric oxice
OPC: oligodendrocyte precursor cell
PD: Parkinson's disease
PDE: phosphodiesterase
PKA: protein kinase A
PKG: protein kinase G
PSD95: postsynaptic density protein 95
RNA: ribonucleic acid
SIRP: signal regulatory protein alpha
SOD1: superoxide dismutase 1
SR-A: scavenger receptor class A
TGF: transforming growth factor beta
TLR4: Toll-like receptor 4
TNFα: tumor necrosis factor α
TREM2: triggering receptor expressed on myeloid cells 2
UCR: upstream conserved region
vGlut1: vesicular glutamate transporter 1
YM-1: chitinase 3-like 3
